# Association between depression and functional autonomy in older adults

**DOI:** 10.1192/j.eurpsy.2026.11588

**Published:** 2026-07-31

**Authors:** S. Omri, N. Bouattour, B. Cherif, I. Gassara, M. Mâalej, R. Feki, M. Mâalej, N. Smaoui, N. Charfi, L. Zouari

**Affiliations:** Psychiatry C, Hedi Chaker University Hospital, Sfax, Tunisia

## Abstract

**Introduction:**

The number of older adults in our society is increasing. Additionally, their medical and mental health problems. Considering the senile evolution of their health, specific care should be taken in order to help them age in a healthy way, in order to keep them independent.

**Objectives:**

To evaluate the relationship between depression and functional autonomy in older adults.

**Methods:**

This cross-sectional, descriptive, and analytical study was conducted in two clinics in the governorate of Sfax between November 1, 2024, and January 31, 2025. Participants included patients aged 60 years or older at the time of the survey, lucid subjects without neurocognitive impairment, and those who provided their free and informed verbal consent. We opted for the Geriatric depression scale (GDS) to assess depression and the Activities of Daily Living (ADL, or the Katz scale) to evaluate a person’s functional autonomy.

**Results:**

Our study included 270 participants. The mean age was 67.33 ± 7.07 years. The sex ratio (M/F) was 0.8. The majority of participants had an educational level not exceeding high school. Married participants represented 73% (n=197). The socioeconomic status was low in 28.9% of cases (n=78). Among the participants, 68.9% (n=186) had a history of chronic somatic condition. Twenty-nine participants (10.7%) had definite depression. The mean ADL score was 5.49 ± 1.08, with extremes ranging from 0 to 6. Thirteen-point three percent were in a situation of mild dependence, 13.3% in a situation of moderate dependence, and 0.4% in a situation of severe dependence. The GDS scale score was significantly higher in subjects with loss of functional autonomy (5.15 vs. 4.15; p=0.046).

**Conclusions:**

Maintaining a functional autonomy in daily activity was associated with lower scores on the depression scale in older adults, which can be a protective factor against depression. For the matter, promoting better health can help older adults to maintain a healthier style of life and therefore keep functional autonomy.

**Disclosure of Interest:**

None Declared

